# Association of sleep quality with glycemic variability assessed by flash glucose monitoring in patients with type 2 diabetes

**DOI:** 10.1186/s13098-021-00720-w

**Published:** 2021-09-23

**Authors:** Yang Yang, Li-hua Zhao, Dan-dan Li, Feng Xu, Xiao-hua Wang, Chun-feng Lu, Chun-hua Wang, Chao Yu, Xiu-lin Zhang, Li-yan Ning, Xue-qin Wang, Jian-bin Su, Li-hua Wang

**Affiliations:** 1 Department of Nursing, Affiliated Hospital 2 of Nantong University, and First People’s Hospital of Nantong City, No. 6 Haierxiang North Road, Nantong, 226001 China; 2 Department of Endocrinology, Affiliated Hospital 2 of Nantong University, and First People’s Hospital of Nantong City, No. 6 Haierxiang North Road, Nantong, 226001 China; 3 Department of Clinical Laboratory, Affiliated Hospital 2 of Nantong University, and First People’s Hospital of Nantong City, No. 6 Haierxiang North Road, Nantong, 226001 China; 4 Department of Administration, Affiliated Hospital 2 of Nantong University, and First People’s Hospital of Nantong City, No.6 Haierxiang North Road, Nantong, 226001 China

**Keywords:** Glycemic variability, Sleep quality, Type 2 diabetes

## Abstract

**Background:**

Deterioration of sleep quality has been reported to contribute to the incidence of diabetes and may be responsible for glycemic status in diabetes. The present study explored the relationship between sleep quality and glycemic variability in patients with type 2 diabetes (T2D).

**Methods:**

We recruited 111 patients with T2D for this cross-sectional study. Each patient underwent flash glucose monitoring for 14 days to obtain glycemic variability parameters, such as standard deviation of glucose (SD), coefficient of variation of glucose (CV), mean amplitude of glycemic excursions (MAGE), mean of daily differences (MODD), and time in glucose range of 3.9–10 mmol/L (TIR_3.9–10_). After 14 days of flash glucose monitoring, each patient received a questionnaire on the Pittsburgh Sleep Quality Index (PSQI) to evaluate subjective sleep quality. HbA1c was also collected to assess average glucose.

**Results:**

HbA1c was comparable among the subgroups of PSQI score tertiles. Across ascending tertiles of PSQI scores, SD, CV and MAGE were increased, while TIR_3.9–10_ was decreased (*p* for trend  <  0.05), but not MODD (*p* for trend  =  0.090). Moreover, PSQI scores were positively correlated with SD, CV, MODD and MAGE (*r* =  0.322, 0.361, 0.308 and 0.354, respectively,* p*  <  0.001) and were inversely correlated with TIR_3.9–10_ (*r*  =  − 0.386, *p*  <  0.001). After adjusting for other relevant data by multivariate linear regression analyses, PSQI scores were independently responsible for SD (*β*  =  0.251,* t*  =  2.112, *p*  =  0.041), CV (*β*  =  0.286,* t*  =  2.207, *p*  =  0.033), MAGE (*β*  =  0.323, *t*  =  2.489, *p*  =  0.018), and TIR_3.9–10_ (*β*  =  − 0.401,* t*  =  − 3.930, *p*  <  0.001) but not for MODD (*β*  =  0.188,* t * =  1.374, *p*  =  0.177).

**Conclusions:**

Increased glycemic variability assessed by flash glucose monitoring was closely associated with poor subjective sleep quality evaluated by the PSQI in patients with T2D.

**Supplementary Information:**

The online version contains supplementary material available at 10.1186/s13098-021-00720-w.

## Background

Glycemic variability, defined as the instability between high and low values of glycemia [1], has been demonstrated to stimulate oxidative stress and provoke proinflammatory mediators [2–4], which in turn lead to various vascular complications in patients with type 2 diabetes (T2D) [5–7]. Moreover, glycemic variability is independent of average glucose levels reflected by glycosylated hemoglobin A1c (HbA1c), and diabetic patients with comparable HbA1c may present with different features of glycemic variability [8, 9] and subsequent diabetic complications [10]. At present, ongoing research efforts worldwide are trying to screen intrinsic and external risk factors for increased glycemic variability, which can help guide the development of appropriate therapeutic regimens to improve glycemic variability and subsequent diabetic prognosis.

Currently, several technologies are available to quantitatively assess all-day glycemic variability, such as continuous glucose monitoring (CGM) systems (such as systems from Medtronic MiniMed Inc.) for 3–7 days and flash glucose monitoring (FGM) systems (such as the systems from Abbott Inc.) for 14 days, which can present a full range of glycemic variability in any time period [11, 12]. A fully detailed profile of glycemic variability may facilitate screening the risk factors for increased glycemic variability.

Sleep is a necessary part of human daily life, and a good quality of sleep is essential to human physical and mental recovery after exhausting work. Conversely, deterioration in sleep quality was reported to account for a wide spectrum of adverse health outcomes, such as chronic metabolic diseases, malignant tumors, adverse cardiovascular events, and all-cause mortality [13–16]. Accordingly, in several previous studies, poor sleep quality was demonstrated to contribute to glycemic disturbances and the occurrence and progression of diabetes [17, 18]. Thus, we hypothesized that poor sleep quality may be an important risk factor for increased glycemic variability. The Pittsburgh Sleep Quality Index (PSQI) is a potent tool to assess subjective sleep quality and is widely applied in sleep studies [19, 20].

Therefore, our present study was conducted to determine whether there was a possible relationship between subjective sleep quality assessed by the PSQI and glycemic variability indices acquired from the FGM system in T2D patients.

## Methods

### Participant recruitment

We released a notification to recruit participants for this study from the Department of Endocrinology, Affiliated Hospital 2 of Nantong University, between December 2019 and January 2021. The inclusion criteria for participants were as follows: (1) aged from 25 to 70 years; (2) diagnosis of T2D referring to the statement released by the American Diabetes Association in 2015 [21]; and (3) consent to join the study. The exclusion criteria for participants were as follows: (1) type 1 diabetes or presence of diabetes-related antibodies; (2) history of malignancy; (3) chronic obstructive pulmonary disease; (4) severe cardio-cerebrovascular diseases, such as myocardial and cerebral infarction; (5) chronic liver and kidney diseases; (6) severe obstructive sleep apnea syndrome; (7) hyperthyroidism or hypothyroidism; (8) current use of glucocorticoids; (9) serious psychiatric diseases; and (10) connective tissue diseases. Ultimately, 111 eligible patients with full data were pooled for statistical analyses. The study was reviewed and approved by the Ethics Committee of Affiliated Hospital 2 of Nantong University, and was conducted in accordance with the Declaration of Helsinki. In addition, all participants provided informed consent when they were recruited into the study.

### Clinical data collection

Clinical data of participants were collected when they were screened by medical history, physical examination and biochemical tests. Relevant data for the final analysis included age, sex, waist circumference (WC), height, weight, systolic blood pressure (SBP), diastolic blood pressure (DBP), history of hypertension and glucose-lowering therapies. Body mass index (BMI) was calculated based on weight and height (kg/m^2^). Hypertension was defined as we described in our previous study [22]. Glucose-lowering therapies in our study were classified into lifestyle alone, insulin treatments, insulin secretagogues, metformin, pioglitazone, α-glucosidase inhibitors (AGIs), dipeptidyl peptidase-4 inhibitors (DPP-4Is) and sodium-glucose cotransporter-2 inhibitors (SGLT-2Is).

Fasting blood samples were drawn to assess triglycerides (TG), total cholesterol (TC), high-density lipoprotein cholesterol (HDLC), low-density lipoprotein cholesterol (LDLC), creatinine (Cr), uric acid (UA) and glycosylated hemoglobin (HbA1c). The estimated glomerular filtration rate (eGFR) was acquired using the Modification of Diet in Renal Disease (MDRD) equation [23].

All patients also received a 75-g oral glucose tolerance test (OGTT) for the assessment of α-cell and β-cell functions. Venous blood samples were drawn at 0, 30, 60, 120, and 180 min after glucose loading to synchronously determine serum glucose, C-peptide and glucagon levels. Overall glucose levels were measured by the area under the glucose curve (AUC_glu_). Overall, α-cell and β-cell functions were measured by the area under the glucagon curve (AUC_gluca_) and the area under the C-peptide curve (AUC_cp_), respectively [24, 25].

### Assessment for glycemic variability

After initial screening, eligible patients with T2D were detected by a flash continuous glucose monitoring (FGM) system for 14 days. The FGM system we used in the present study is a hospital version (FreeStyle™ Libre H, Abbott Diabetes Care Ltd., Witney, Oxon, UK), which contains three parts: a sensor kit, a reader and a software for downloading glucose data from the reader. During the FGM, the individualized plan for daily dietary energy intake was prepared by nutritionists, which was also described in our previous study [22]. The sensor kit can store glucose data at 15-min intervals. To make sensor’s glucose data blind to the patients during the FGM, a reader was not provided to the patients. The sensor’s glucose data were unblinded to the patients after they completed the PSQI questionnaire on the last day of FGM. After 14 days of FGM, glucose data could be downloaded from the reader by the FGM software, the software then generated ambulatory glucose profile (AGP) reports [26], and the time in glucose range from 3.9 to 10 mmol/L (TIR_3.9–10_) could be obtained from the AGP reports. Other multiple glycemic variability indices, including standard deviation of glucose (SD), coefficient of variation of glucose (CV), mean of daily differences (MODD), and mean amplitude of glycemic excursions (MAGE), could be calculated from the downloaded glucose data. The methods of calculation were also described in our previous studies [22, 27] and other studies [7, 28].

### Subjective measurement for sleep quality

After 14 days of FGM, all participants received a Chinese version of the PSQI questionnaire by face-to-face interviews to measure subjective sleep quality [29, 30]. The PSQI, a self-evaluation of sleep quality in the past month, has 19 items, 9 questions and 7 component scales. The 7 components are listed below: (1) subjective sleep quality; (2) sleep latency; (3) sleep duration; (4) sleep efficiency; (5) sleep disturbances; (6) use of sleep medication; and (7) daytime dysfunction. Each component is rated equally on a 0–3 scale, and the 7 components are then pooled to generate total PSQI scores ranging from 0 to 21. Higher PSQI scores represent poorer quality of sleep, which indicates that 0 is better than 21. The Chinese version of the PSQI had an overall reliability coefficient of 0.82–0.83 and an acceptable test–retest reliability coefficient of 0.85 for all subjects [30].

### Statistical analyses

Clinically relevant data of recruited patients with T2D are exhibited for the total and three subgroups according to the tertiles of PSQI scores. Continuous and categorical data are expressed as the mean  ±  standard deviation and frequency (percentage), respectively. We used one-way analysis of variance (ANOVA) with a linear trend to explore trends in continuous data among PSQI score tertiles and used the chi-squared test with linear-by-linear association to explore trends in categorical data among PSQI score tertiles. In addition, we used Pearson’s correlation analysis to explore the correlation of PSQI scores with multiple glycemic variability parameters.

Furthermore, multivariate linear regression analysis was used to adjust for other clinically relevant variables to explore the independent effects of PSQI scores on multiple glycemic variability parameters. In each regression analysis, the initial Model 0 was unadjusted; Model 1 was adjusted for age, sex, BMI, WC, SBP, DBP and diabetes duration; Model 2 was further adjusted for hypertension and glucose-lowering therapies; and Model 3 was further adjusted for eGFR, UA, lipid profiles, HbA1c, AUC_glu_, AUC_cp_ and AUC_gluca_.

We used SPSS for Windows, standard version 19.0 (IBM Co., Armonk, NY, USA), to input and analyze the data. Statistical significance was determined by a *p* value  <  0.05.

## Results

### Clinical characteristics of participants

The clinical characteristics of the recruited patients with T2D are displayed in Table 1. The mean PSOI score of all recruited patients was 6.9 ±  2.9, and the range of PSOI scores was 1–16. The ranges of the PSQI score tertiles were 1–4 (first tertile, T1), 5–7 (second tertile, T2) and 8–16 (third tertile, T3). From T1, T2, to T3 of PSQI scores, SD, CV and MAGE were notably increased, while TIR_3.9–10_ was decreased (*p* for trend  <  0.05), but not MODD (*p* for trend  =  0.090). Moreover, across ascending tertiles of PSQI scores, TC levels were significantly increased, but age, ratio of females, BMI,WC, SBP, DBP, diabetes duration, hypertension prevalence, TG, HDLC, LDLC, UA, eGFR, AUC_glu_, AUC_cp_, AUC_gluca_ and HbA1c did not show any differences among the tertiles of PSQI scores. Regarding glucose-lowering therapies, the frequency of metformin use was increased when the tertiles of PSQI scores increased, but lifestyle alone, insulin treatments, insulin secretagogues, AGIs, DPP-4Is and SGLT-2Is were comparable among the tertiles of PSQI scores.

**Table 1:** Clinical characteristics of the total patients and subgroups based on the tertiles of PSQI scores

Variables	Total	Tertiles of PSOI scores			*F/x*^2^ value	*p* for trend
		T1	T2	T3		
*n*	111	25	41	45	–	–
PSOI scores (range)	6.9 ± 2.9 (1–16)	3.5 ± 0.8 (1–4)	5.8 ± 0.8 (5–7)	9.9 ± 2.0 (8–16)	–	–
Age (year)	50.1 ± 11.1	49.4 ± 9.8	49.4 ± 9.6	51.0 ± 10.7	0.315	0.730
Female, *n *(%)	40 (36.0)	11 (44.0)	13 (31.7)	16 (35.6)	0.315	0.574
BMI (kg/m^2^)	25.3 ± 3.3	26.0 ± 3.2	24.5 ± 2.9	25.6 ± 3.6	1.835	0.165
WC (cm)	89.9 ± 10.5	91.9 ± 8.5	87.8 ± 10.6	90.8 ± 11.2	1.453	0.238
SBP (mmHg)	127.6 ± 14.4	124.6 ± 15.3	130.1 ± 15.3	126.9 ± 13.1	1.193	0.307
DBP (mmHg)	79.8 ± 9.3	79.4 ± 10.7	79.9 ± 9.6	80.0 ± 8.5	0.043	0.958
Diabetes duration (year)	1.73 ± 1.02	1.56 ± 0.87	1.80 ± 1.03	1.76 ± 1.09	0.470	0.627
Glucose-lowering therapies						
Lifestyle alone, *n *(%)	10 (9.0)	1 (4.0)	3 (7.3)	6 (13.3)	1.862	0.172
Insulin treatments, *n *(%)	29 (26.1)	4 (16.0)	10 (24.4)	15 (33.3)	2.579	0.108
Insulin-secretagogues, *n *(%)	17 (15.3)	4 (16.0)	6 (14.6)	7 (15.6)	0.001	0.983
Metformin, *n *(%)	75 (67.6)	21 (84.0)	28 (68.3)	26 (57.8)	4.937	0.026
Pioglitazone, *n *(%)	12 (10.8)	3 (12.0)	4 (9.8)	5 (11.1)	0.004	0.949
AGIs, *n *(%)	6 (5.4)	3 (12.0)	2 (4.9)	1 (2.2)	2.771	0.096
DPP-4Is, *n *(%)	3 (2.7)	1 (4.4)	1 (2.4)	1 (2.2)	0.166	0.684
SGLT-2Is, *n *(%)	15 (13.5)	5 (20.0)	4 (9.8)	6 (13.3)	0.370	0.543
Hypertension, *n *(%)	45 (40.5)	8 (32.0)	16 (39.0)	21 (46.7)	1.482	0.224
TG (mmol/L)	2.57 ± 2.53	3.52 ± 4.19	2.40 ± 1.74	2.13 ± 1.43	1.997	0.143
TC (mmol/L)	4.57 ± 1.04	5.09 ± 1.01	4.37 ± 1.06	4.42 ± 0.95	4.529	0.034
HDLC (mmol/L)	1.14 ± 0.28	1.11 ± 0.25	1.11 ± 0.31	1.19 ± 0.27	0.697	0.501
LDLC (mmol/L)	2.96 ± 0.93	3.31 ± 0.86	2.79 ± 0.89	2.89 ± 0.97	2.048	0.136
UA (μmol/L)	332.1 ± 92.0	336.3 ± 91.3	327.6 ± 113.7	333.4 ± 74.1	0.055	0.947
eGFR (mL/min/1.73m^2^)	164.7 ± 32.9	174.8 ± 35.1	161.5 ± 29.0	161.5 ± 34.2	1.241	0.295
AUC_glu_ (mmol/L·h)	42.9 ± 8.9	44.0 ± 7.1	41.9 ± 8.9	43.3 ± 10.1	0.401	0.671
AUC_cp_ (ng/mL·h)	13.8 ± 4.4	14.1 ± 4.1	13.5 ± 4.8	13.9 ± 4.2	0.159	0.853
AUC_gluca_ (pg/mL·h)	464.4 ± 185.3	481.7 ± 240.0	430.7 ± 144.1	485.1 ± 180.9	0.888	0.415
HbA1c (%)	7.73 ± 1.54	7.35 ± 1.29	7.75 ± 1.58	7.92 ± 1.63	1.087	0.341
SD (mmol/L)	2.62 ± 0.79	2.32 ± 0.63	2.67 ± 0.73	2.73 ± 0.88	4.010	0.048
CV (%)	34.5 ± 6.9	31.3 ± 6.6	35.1 ± 6.2	35.7 ± 7.2	5.897	0.017
MAGE (mmol/L)	5.85 ± 1.25	5.24 ± 1.03	5.94 ± 1.15	6.09 ± 1.36	6.849	0.010
MODD (mmol/L)	2.13 ± 0.69	1.93 ± 0.56	2.14 ± 0.70	2.23 ± 0.74	2.923	0.090
TIR_3.9–10_ (%)	72.0 ± 12.4	78.1 ± 9.8	71.9 ± 12.0	68.6 ± 12.9	9.837	0.002

Continuous data are expressed as mean  ±  standard deviation, and categorical data are expressed as frequency (percentage)

ANOVA with a linear trend and chi-squared test with linear-by-linear association were applied to detect trends in continuous data and categorical data among tertiles of PSQI scores, respectively

*PSQI* Pittsburgh Sleep Quality Index; *BMI* body mass index; *WC* waist circumference; *SBP*/*DBP* systolic/diastolic blood pressure; *AGIs* α-glucosidase inhibitors; *DPP*-*4Is* dipeptidyl peptidase-4 inhibitors; *SGLT*-*2Is* sodium-glucose cotransporter-2 inhibitors; *TG* triglycerides; *TC* total cholesterol; *HDLC* high-density lipoprotein cholesterol; *LDLC* low-density lipoprotein cholesterol; *UA* uric acid; *HbA1c* glycosylated hemoglobin A1c; *eGFR* estimated glomerular filtration rate; *AUC*_*glu*_ area under the glucose curve; *AUC*_*gluca*_ area under the glucagon curve; *AUC*_*cp*_ area under the C-peptide curve; *SD* standard deviation of glucose; *CV* coefficient of variation of glucose; *MAGE* mean amplitude of glycemic excursions; *MODD* mean of daily differences; *TIR*_*3*.9–10_ time in glucose range of 3.9–10 mmol/L

### Correlations between PSQI scores and multiple glycemic variability parameters

**Fig. 1:**
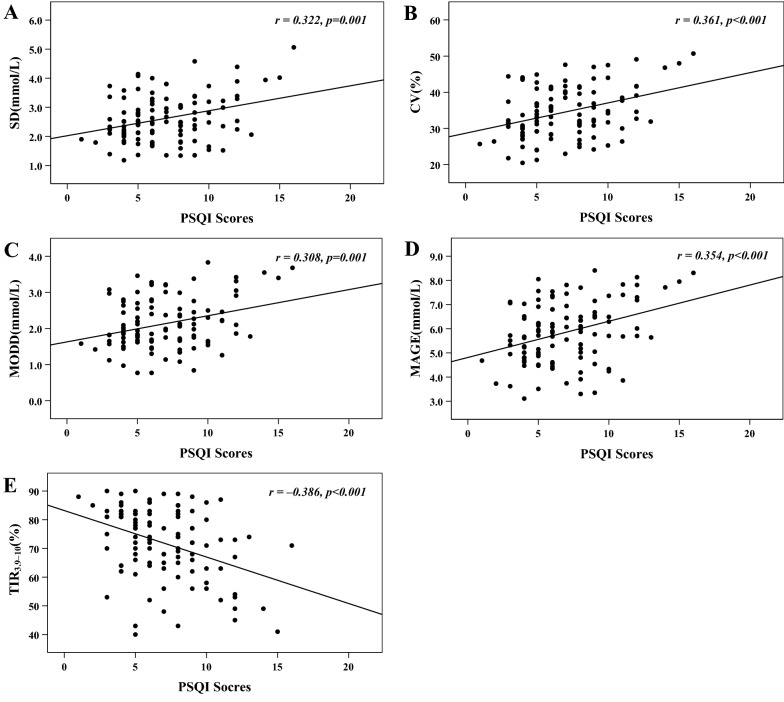
Scatter plots for the correlations of multiple glycemic variability parameters with PSQI scores. **A** standard deviation of glucose (SD); **B** coefficient of variation of glucose (CV); **C** mean of daily differences (MODD); **D** mean amplitude of glycemic excursions (MAGE); **E** time in glucose range of 3.9–10 mmol/L (TIR_3.9–10_)

Pearson’s correlation analysis showed that SD, CV, MODD and MAGE were positively correlated with PSQI scores (*r*  =  0.322, 0.361, 0.308 and 0.354, respectively,* p * <  0.001), while TIR_3.9–10_ was negatively correlated with PSQI scores (*r*  =  − 0.386, *p*  <  0.001). These correlations are also graphically displayed in Fig. 1. Additionally, the corrections of the 7 components in the PSQI with multiple glycemic variability parameters are shown in Additional file 1: Table S1. Overall, subjective sleep quality, sleep latency, sleep duration, sleep efficiency and daytime dysfunction were related to glycemic variability parameters.

### Multivariate linear regression analyses exploring the independent effects of PSQI scores on multiple glycemic variability parameters

The effects of the PSQI scores on the outcomes of multiple glycemic variability parameters by multivariate linear regression analyses are shown in Table 2. In the initial unadjusted Model 0, PSQI scores were independently associated with SD (*β*  =  0.322,* t*  =  3.551, *p*  =  0.001, adjusted *R*^2^ = 0.104), CV (*β*  =  0.361,* t*  =  4.307, *p*  <  0.001, adjusted *R*^2^ = 0.130), MODD (*β*  =  0.308,* t*  =  3.381, *p * =  0.001, adjusted *R*^2^ = 0.095), MAGE (*β*  =  0.354,* t*  =  3.948, *p*  <  0.001, adjusted *R*^2^ = 0.125), and TIR_3.9–10_ (*β*  =  − 0.386,* t*  =  − 4.373, *p*  <  0.001, adjusted *R*^2^ = 0.149). After adjusting for other clinically relevant variables by multivariate linear regression analyses, the adjusted *R*^*2*^ was revealed to gradually increase from Model 0 to Model 3. In fully adjusted Model 3, PSQI scores remained independently associated with SD (*β*  =  0.251,* t*  =  2.112, *p * =  0.041), CV (*β*  =  0.286,* t*  =  2.207, *p*  =  0.033), MAGE (*β*  =  0.323,* t * =  2.489, *p*  =  0.018), and TIR_3.9–10_ (*β*  =  − 0.401,* t*  =  − 3.930, *p*  <  0.001) but not with MODD (*β*  =  0.188,* t*  =  1.374, *p*  =  0.177).

**Table 2:** Multiple linear regression models displaying the effects of PSQI scores on the outcomes of glycemic variability parameters

Models	B (95% CI)	*β*	*t*	*p*	Adjusted *R*^2^
SD					
Model 0: unadjusted	0.086 (0.038–0.134)	0.322	3.551	0.001	0.104
Model 1: age, sex, BMI, WC, SBP, DBP and diabetes duration	0.080 (0.031–0.129)	0.300	3.231	0.002	0.145
Model 2: Model 1 + hypertension and glucose-lowering therapies	0.060 (0.014–0.106)	0.225	2.588	0.011	0.366
Model 3: Model 2 + eGFR, UA, lipid profiles, HbA1c, AUC_glu_, AUC_cp_ and AUC_gluca_	0.065 (0.003–0.128)	0.251	2.112	0.041	0.678
CV					
Model 0: unadjusted	0.838 (0.427–1.250)	0.361	4.037	< 0.001	0.130
Model 1: age, sex, BMI, WC, SBP, DBP and diabetes duration	0.810 (0.391–1.229)	0.348	3.832	< 0.001	0.179
Model 2: Model 1 + hypertension and glucose-lowering therapies	0.676 (0.254–1.099)	0.291	3.177	0.002	0.295
Model 3: Model 2 + eGFR, UA, lipid profiles, HbA1c, AUC_glu_, AUC_cp_ and AUC_gluca_	0.667 (0.055–1.279)	0.286	2.207	0.033	0.618
MODD					
Model 0: unadjusted	0.072 (0.030–0.115)	0.308	3.381	0.001	0.095
Model 1: age, sex, BMI, WC, SBP, DBP and diabetes duration	0.065 (0.022–0.109)	0.279	2.999	0.003	0.143
Model 2: Model 1 + hypertension and glucose-lowering therapies	0.050 (0.009–0.091)	0.213	2.402	0.018	0.342
Model 3: Model 2 + eGFR, UA, lipid profiles, HbA1c, AUC_glu_, AUC_cp_ and AUC_gluca_	0.041 (–0.019 to 0.100)	0.188	1.374	0.177	0.575
MAGE					
Model 0: unadjusted	0.150 (0.075–0.225)	0.354	3.948	< 0.001	0.125
Model 1: age, sex, BMI, WC, SBP, DBP and diabetes duration	0.138 (0.061–0.215)	0.326	3.563	0.001	0.169
Model 2: Model 1 + hypertension and glucose-lowering therapies	0.113 (0.038–0.187)	0.266	3.004	0.003	0.342
Model 3: Model 2 + eGFR, UA, lipid profiles, HbA1c, AUC_glu_, AUC_cp_ and AUC_gluca_	0.135 (0.025–0.246)	0.323	2.489	0.018	0.614
TIR_3.9–10_					
Model 0: unadjusted	− 1.619 (− 2.353 to − 0.885)	− 0.386	− 4.373	< 0.001	0.149
Model 1: age, sex, BMI, WC, SBP, DBP and diabetes duration	− 1.597 (− 2.345 to − 0.849)	− 0.381	− 4.236	< 0.001	0.196
Model 2: Model 1 + hypertension and glucose-lowering therapies	− 1.339 (− 2.076 to − 0.602)	− 0.319	− 3.607	< 0.001	0.347
Model 3: Model 2 + eGFR, UA, lipid Profiles, HbA1c, AUC_glu_, AUC_cp_ and AUC_gluca_	− 1.701 (− 2.577 to − 0.825)	− 0.401	− 3.930	< 0.001	0.763

*PSQI* Pittsburgh Sleep Quality Index; *SD* standard deviation of glucose; *CV* coefficient of variation of glucose; *MAGE* mean amplitude of glycemic excursions; *MODD* mean of daily differences; *TIR*_*3.9–10*_ time in glucose range of 3.9–10 mmol/L; *BMI* body mass index; *WC* waist circumference; *SBP*/*DBP* systolic/diastolic blood pressure; *UA* uric acid; *HbA1c* glycosylated hemoglobin A1c; *eGFR* estimated glomerular filtration rate; *AUC*_*glu*_ area under the glucose curve; *AUC*_*gluca*_ area under the glucagon curve; *AUC*_*cp*_ area under the C-peptide curve

## Discussion

In the present study, we systemically analyzed the relationship between sleep quality assessed by PSQI scores and glycemic variability assessed by FGM in 111 patients with T2D. The main findings of our study were as follows: first, PSQI scores were closely correlated with multiple glycemic variability parameters by univariate analysis, including SD, CV, MODD, MAGE and TIR_3.9–10_; second, PSQI scores were independently associated with SD, CV, MAGE and TIR_3.9–10_ by multivariate linear regression analysis, but not with MODD; third, HbA1c was comparable among the subgroups of PSQI score tertiles, which may suggest that poor subjective sleep quality may have effects on glycemic variability, but not on HbA1c; fourth, after adjusting for other relevant clinical data, each one-point increment in PSQI scores may correspond to an SD increase of 0.251 mmol/L, a CV increase of 0.286%, a MAGE increase of 0.323 mmol/L and a TIR_3.9–10_ decrease of 0.401%.

### Classical glycemic variability parameters and adverse consequences

It is well known that increased glycemic variability is independently associated with a variety of adverse outcomes [31]. Classical glycemic variability parameters, calculated from detailed glycemic profiles obtained by CGM or FGM, including SD, CV, MODD, MAGE and TIR_3.9–10_, have been widely applied in clinical studies. Glycemic variability has its own independent potential to prompt oxidative stress and subsequent adverse health outcomes. As early as 2006, Monnier et al. [4] demonstrated that MAGE was closely associated with urinary 8-iso prostaglandin F_2α_ in patients with T2D, which indicated that glycemic variability could induce a special effect on oxidative stress and paved the road linking glycemic variability to diabetic complications. Ohara et al. [3] also demonstrated that day-to-day glycemic variability assessed by MODD was related to diacron-reactive oxygen metabolites reflective of oxidative stress. Moreover, with respect to diabetic complications, SD was recognized as a significant risk factor for diabetic retinopathy in patients with pooled type 1 diabetes and T2D [32], and CV was closely connected to the prevalence of cardiovascular autonomic neuropathy in patients with T2D [7]. In addition, increased MAGE was not only found to be associated with the presence and severity of coronary artery disease in patients with T2D but could also predict major adverse cardiovascular events in patients who had experienced acute myocardial infarction [5, 33]. Furthermore, TIR_3.9–10_ has been the center of much attention in recent years. Decreased TIR_3.9–10_ was revealed to be associated with diabetic retinopathy [34], painful diabetic polyneuropathy [35], impaired peripheral nerve functions [36], increased carotid intima-media thickness [37] and cardiovascular autonomic neuropathy [38] in patients with T2D. Therefore, screening modifiable risk factors for increased glycemic variability would be of significance.

### Possible risk factors for increased glycemic variability

Accumulated studies have revealed the intrinsic and external risk factors for increased glycemic variability. Our previous studies have shown that impaired islet β-cell function may account for increased glycemic variability in subjects at high risk for diabetes and in patients with T2D [39, 40], which was consistent with a prior study by Kohnert et al. [41] in T2D using oral hypoglycemic agents. Correspondingly, glycemic variability could be attenuated by improvement in β-cell function [42]. Moreover, increased glycemic variability may also be related to lower levels of fasting C-peptide, longer diabetic duration in T2D using insulin, older age, obesity, higher TG, lower LDLC and the use of sulfonylurea in T2D without insulin treatment [43]. In addition, abnormal glucagon secretion [44], hyperthyroidism [45], higher serum thyrotropin [22], more severe dawn phenomenon [46] and acute stress conditions [47] could prompt glycemic variability. Furthermore, in our present study, poor subjective sleep quality estimated by the PSQI may lead to deterioration in glycemic variability.

### Poor sleep quality and adverse consequences

Normal sleep is a physiological process for energy restoration and replenishment and serves a reparative role in physical and mental fatigue relief. Normal sleep is characterized by decreases in glucose turnover and metabolic demand. Sleep inefficiency or poor sleep quality was demonstrated to be responsible for obesity [48], hypertension [49], type 2 diabetes [50], gestational diabetes mellitus [51], cardiovascular disease [14], and prognosis of chronic diseases [52]. In our present study, we found that multiple glycemic variability parameters, such as SD, CV, MODD, MAGE and TIR_3.9–10_, were correlated well with subjective sleep quality. In the final analysis, subjective sleep quality may independently account for SD, CV, MAGE and TIR_3.9–10_. More surprisingly, HbA1c was comparable among the subgroups of PSQI score tertiles. These results suggested that poor subjective sleep quality may have effects on glycemic variability but not on HbA1c. Approaches to improve sleep quality may facilitate the amelioration of glycemic variability.

### Underlying mechanism for the linkage of sleep quality to glycemic variability

Several studies have suggested multiple pathways in the possible connection between poor sleep quality and increased glycemic variability. These pathways involve impaired cerebral glucose utilization, a hyperactive sympathetic system, the release of proinflammatory cytokines, rhythmic alterations in cortisol and growth hormone secretion, abnormalities in adipocyte function and dysregulation in appetite-regulating hormones [53, 54]. During the period of sleep deprivation, cerebral glucose utilization was revealed to be markedly reduced, notably in some cortical and subcortical regions [55]. After poor sleep quality, the sympathetic nervous system is overactivated, which in turn leads to insulin resistance and aberrant glucagon secretion [54, 56]. Consistently, elevation of systemic inflammatory responses in relation to sleep restriction was also well demonstrated, and proinflammatory cytokines such as tumor necrosis factor and C-reactive protein were released and subsequently promoted insulin resistance [57, 58]. Moreover, increased cortisol secretion in the afternoon and evening and prolonged growth hormone secretion at night due to sleep restriction could also facilitate insulin resistance [59, 60]. Additionally, abnormalities in adipocyte function were proven to be connected to adverse metabolic consequences after poor sleep quality. Sleep restriction may account for a 30% reduction in the efficiency of the insulin signaling pathways in adipocytes [61]. Furthermore, appetite-regulating hormones were found to be dysregulated during sleep restriction; for example, leptin, a satiety hormone, was decreased, while ghrelin, a hunger hormone, was increased [62]. These changes in appetite-regulating hormones may be responsible for increases in food intake and body mass and subsequent insulin resistance. Therefore, as a result of insufficient sleep, numerous risk factors for the above suggestive pathways could cross-promote with each other, induce insulin resistance, and facilitate islet β-cell dysfunction and the incidence of T2D, ultimately contributing to the increased glycemic variability.

### Strengths

Our present study exhibits several strengths. First, our study may be the first to explore the relationship between subjective sleep quality assessed by PSQI and glycemic variability assessed by FGM in patients with T2D. Second, the FGM data were blinded to the patients during FGM detection. Third, multiple glycemic variability parameters were applied in our present study, especially TIR_3.9–10_, which has received extensive attention because of its central role in diabetic complications.

### Limitations

Several limitations of our present study should be recognized. First, our study was cross-sectionally conducted and may not conclude a causal relationship between poor sleep quality and increased glycemic variability. A longitudinal study must be performed to compensate for this defect. Second, our study is restricted to the Chinese population with T2D in a single center, and the findings have limited generalizability. Third, the PSQI is a self-report measurement to subjectively assess sleep quality over the previous month. Our study lacks an objective measure for sleep, such as polysomnography (PSG), but PSG is restricted to monitoring sleep for only one or two nights. It would be ideal to combine PSQI with PSG for sleep studies.

## Conclusions

In summary, increased glycemic variability assessed by FGM was closely associated with poor sleep quality assessed by the PSQI score in patients with T2D, which indicated that clinical strategies targeting improving sleep quality may ameliorate glycemic variability.

## Supplementary Information

**Additional file 1: Table S1. **Relationships between the 7 components of the PSQI and glycemic variability parameters in patients with T2D.

## Data Availability

The current data are available to all interested researchers upon reasonable request. Requests for access to data should be made to the principal investigators of the study.

## Abbreviations

T2D: Type 2 diabetes
FGM: Flash glucose monitoring
CGM: Continuous glucose monitoring
SD: Standard deviation of glucose
CV: Coefficient of variation of glucose
MAGE: Mean amplitude of glycemic excursions
MODD: Mean of daily differences
TIR_3.__9–10_: Time in glucose range of 3.9–10 mmol/L
PSQI: Pittsburgh Sleep Quality Index
WC: Waist circumference
SBP/DBP: Systolic/diastolic blood pressure
BMI: Body mass index
AGIs: α-Glucosidase inhibitors
DPP-4Is: Dipeptidyl peptidase-4 inhibitors
SGLT-2Is: Sodium-glucose cotransporter-2 inhibitors
TG: Triglycerides
TC: Total cholesterol
HDLC: High-density lipoprotein cholesterol
LDLC: Low-density lipoprotein cholesterol
Cr: Creatinine
UA: Uric acid
HbA1c: Glycosylated hemoglobin A1c
eGFR: Estimated glomerular filtration rate
OGTT: Oral glucose tolerance test
AUC_glu_: Area under the glucose curve
AUC_gluca_: Area under the glucagon curve
AUC_cp_: Area under the C-peptide curve
