# Supplementary material for: Association of sleep quality with glycemic variability assessed by flash glucose monitoring in patients with type 2 diabetes
Source: Diabetol Metab Syndr. 2021 Sep 23;13:102. doi: 10.1186/s13098-021-00720-w (PMC8461905; doi:10.1186/s13098-021-00720-w)
Supplement: Supplementary file 1 — Additional file 1: Table S1. Relationships between the 7 components of the PSQI and glycemic variability parameters in patients with T2D. [file 13098_2021_720_MOESM1_ESM.docx]

**Supplementary Table 1** Relationships between the 7 components of the PSQI and glycemic variability parameters in patients withT2D

|  |  | SD | CV | MODD | MAGE | TIR_3.9–10_ |
| --- | --- | --- | --- | --- | --- | --- |
| PSQI scores | *r* | 0.322 | 0.361 | 0.308 | 0.354 | –0.386 |
|  | *p* | <0.001 | <0.001 | 0.001 | <0.001 | <0.001 |
| Subjective sleep quality | *r* | 0.197 | 0.179 | 0.229 | 0.213 | –0.350 |
|  | *p* | 0.038 | 0.060 | 0.016 | 0.025 | <0.001 |
| Sleep latency | *r* | 0.283 | 0.302 | 0.231 | 0.298 | 0.296 |
|  | *p* | 0.003 | 0.001 | 0.015 | 0.002 | 0.002 |
| Sleep duration | *r* | 0.261 | 0.257 | 0.284 | 0.327 | –0.324 |
|  | *p* | 0.006 | 0.006 | 0.003 | <0.001 | 0.001 |
| Sleep efficiency | *r* | 0.313 | 0.315 | 0.310 | 0.329 | –0.317 |
|  | *p* | 0.001 | 0.001 | 0.001 | 0.001 | 0.001 |
| Sleep disturbances | *r* | 0.056 | 0.124 | 0.028 | 0.055 | –0.101 |
|  | *p* | 0.559 | 0.194 | 0.773 | 0.563 | 0.293 |
| Use of sleep medication | *r* | –0.064 | 0.034 | –0.065 | –0.013 | –0.012 |
|  | *p* | 0.507 | 0.722 | 0.496 | 0.894 | 0.901 |
| Daytime dysfunction | *r* | 0.209 | 0.224 | 0.175 | 0.185 | –0.211 |
|  | *p* | 0.028 | 0.018 | 0.067 | 0.052 | 0.026 |

PSQI: Pittsburgh Sleep Quality Index; SD: standard deviation of glucose; CV: coefficient of variation of glucose; MAGE: mean amplitude of glycemic excursions; MODD: mean of daily differences; TIR_3.9-10_: time in glucose range of 3.9–10 mmol/L.
